# A rare case of repeated anastomotic recurrence due to tumor implantation after curative surgery for sigmoid colon cancer

**DOI:** 10.1186/1477-7819-5-91

**Published:** 2007-08-06

**Authors:** Kimihiko Funahashi, Junichi Koike, Naoyasu Saito, Hiroyuki Shiokawa, Kentaro Shirasaka, Tatsuo Teramoto

**Affiliations:** 1 Division of Gastroenterological Surgery, Toho University Omori Medical Center, Tokyo, Japan

## Abstract

**Background:**

Anastomotic recurrence is often experienced at colocolic or colorectal anastomoses. Tumor cell implantation has been reported as the mechanism of anastomotic recurrence. However, anastomotic recurrence occurring repeatedly after curative surgery is rare. We herein report a rare case of repeated anastomotic recurrence after curative surgery for sigmoid colon cancer.

**Case presentation:**

A 51-year-old man underwent radical surgery for sigmoid colon cancer. However, anastomotic recurrence developed three times during three years and six months after the initial operation in spite of irrigation with 5% povidone-iodine before anastomosis. The serum carcinoembryonic antigen (CEA) level had been within normal limits after sigmoidectomy. Finally, the patient underwent abdominoperineal resection. The clinico-pathological findings revealed that possible tumor cell implantation caused these anastomotic recurrences. The patients survived without recurrence during the follow-up period of seven years and nine months.

**Conclusion:**

We experienced a rare case of repeated anastomotic recurrence due to possible tumor implantation after curative surgery for sigmoid colon cancer; however the prognosis was ultimately very good. CEA monitoring was insensitive for detection of anastomotic recurrence in this case.

## Background

Anastomotic recurrence is likely to develop at colocolic or colorectal anastomoses. The incidence rate is reported at 5–10% [1-3]. However, anastomotic recurrence occurring repeatedly after curative surgery is rare. Here we report a case of anastomotic recurrence that occurred three times within three years and six months after curative surgery of sigmoid colon cancer.

## Case presentation

**Figure 1:**
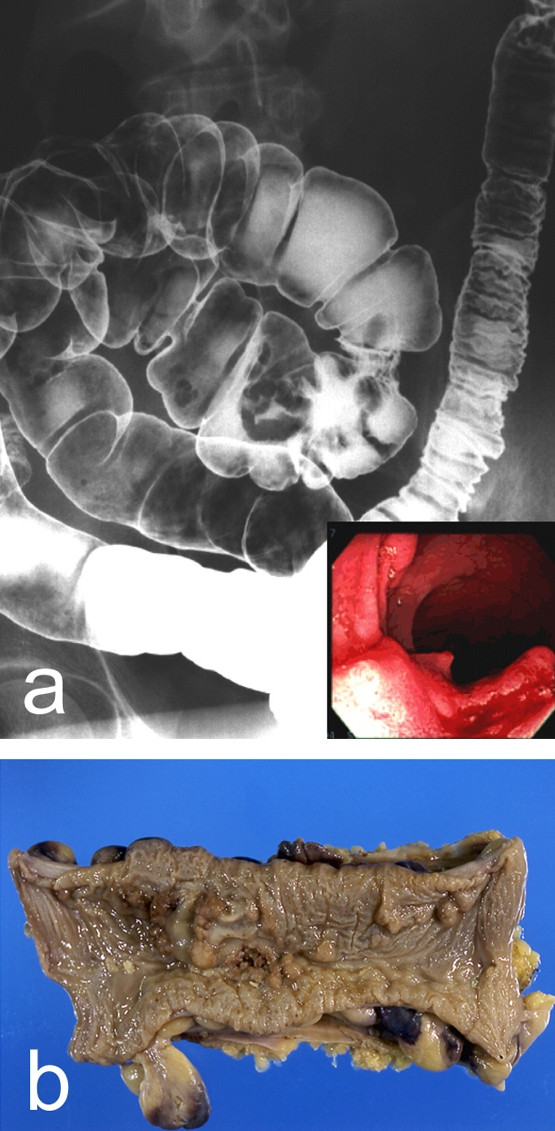
**The primary lesion of the sigmoid colon**. Sigmoid colon cancer was suspected by barium enema and colonoscopy (a). Histological examination revealed the tumor to be a well-differentiated adenocarcinoma invading the subserosa with two lymph nodes metastases (T3, N1, M0). Tumor cells were not identified at the surgical margins (b).

A 51-year-old man visited our hospital with complaint of fecal occult blood. The laboratory data were with-in normal limits (CEA: 2.3 ng/ml, CA19-9: 38 U/ml). Barium enema and colonoscopy showed an elevated lesion with depression and three subpedenculated polyps in the sigmoid colon. Pathological findings on biopsy confirmed a diagnosis of adenocarcinoma. We performed sigmoidectomy with lymphadenectomy in June 1999. Histological examination revealed the tumor to be a well-differentiated adenocarcinoma invading the subserosa with two lymph nodes metastases (T3, N1, M0). Tumor cells were not identified at the surgical margins. One of three subpedenculated polyps was diagnosed histologically as an adenocarcinoma in adenoma, and the others were adenomas (Figure 1).

**Figure 2:**
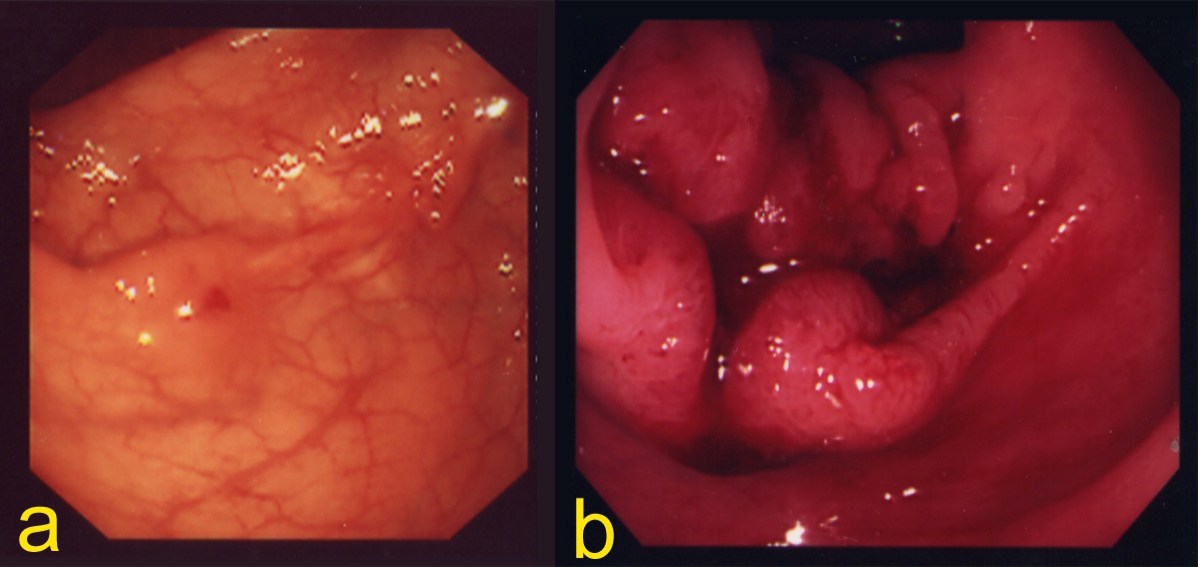
**Follow-up colonoscopy**. We suspected the first anastomotic recurrence by colonoscopy in January 2000. Although an irregular lesion was noticed at the suture line by colonoscopy, pathological examination of the biopsy was unable to confirm malignant cells in the specimen (a). The next colonoscopy, which was performed nine months later, identified an ulcerated tumor occupying the lumen massively (b).

**Figure 3:**
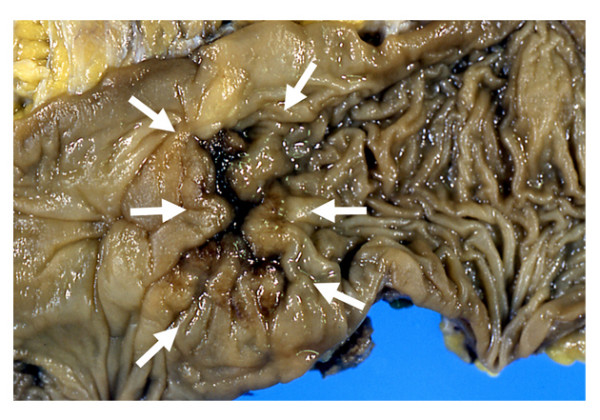
**The first anastomotic recurrence**. The lesion was located in the suture line macroscopically, and was identified as a well-differentiated adenocarcinoma invading the subserosa histologically. There was no metastasis in the regional lymph nodes.

The patient was referred to our hospital by a general practitioner because of fecal occult blood. The first anastomotic recurrence was suspected by colonoscopy in January 2000. Although an irregular lesion was noticed at the suture line by colonoscopy, pathological examination of the biopsy was unable to confirm malignant cells in the specimen. The next colonoscopy, which was performed nine months later, identified an ulcerated tumor occupying the lumen massively (Figure 2). However, both the serum CEA level (2.4 ng/ml) and the CA19-9 level (39 U/ml) were within normal limits at this time. An operation was performed for this lesion in November 2000. We performed anterior resection, and irrigated the intestinal lumen carefully with 5% povidone-iodine before anastomosis to prevent anastomotic recurrence. The lesion was located in the suture line macroscopically, and was identified as a well-differentiated adenocarcinoma invading the subserosa histologically. There was no metastasis in the regional lymph nodes (Figure 3).

**Figure 4:**
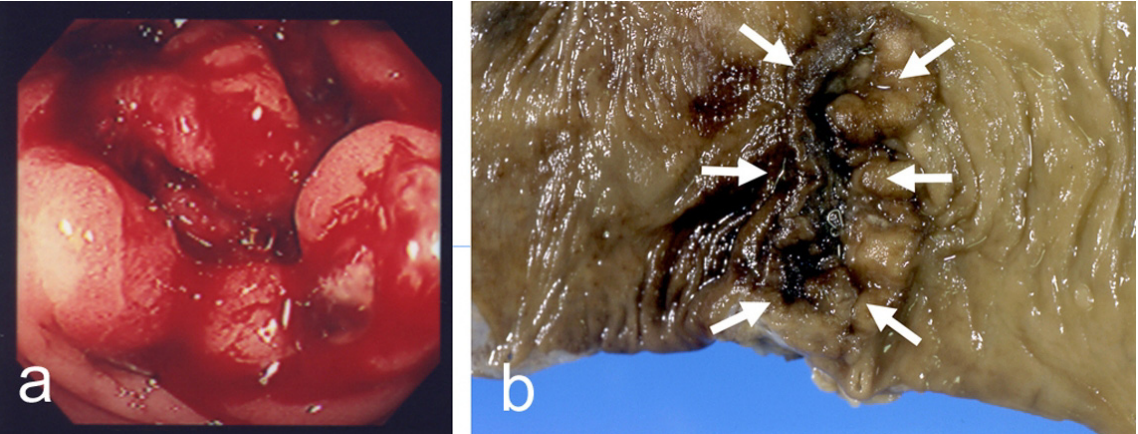
**The second anastomotic recurrence**. Colonoscopy performed in July 2001 showed the second recurrence at the suture line (a). The lesion was located in the suture line macroscopically (arrow). The pathological examination revealed a moderately differentiated adenocarcinoma invading from the muscularis propriae to the submucosal layer in the suture line.

The patient was followed up in the out-patient department with adjuvant chemotherapy of 5-fluorouracil and leucovorin. We suspected recurrence of colon cancer by the elevation of the serum CEA level (6.9 ng/ml) on July 2001, and it was nine months after the prior operation that the second anastomotic recurrence was found. Computed tomography (CT) and ultrasonography (US) did not describe distant organ metastasis or local recurrence in the pelvis, but colonoscopy and barium enema performed in July 2001 showed the second recurrence of disease at the suture line. An operation was performed on December 2001, and the tumor was resected *en bloc *with a part of the small intestine and bladder to which direct invasion was suspected from the preoperative CT. Although metastasis was also suspected in both the peritoneum and the regional lymph nodes around the tumor intra-operatively, tumor cells were not identified in either the peritoneum or the regional lymph nodes by microscopic examination of a frozen-section specimen histologically. We performed a low anterior resection for the second recurrence of disease, and performed anastomosis by the double-stapling technique after irrigation with 5% povidone-iodine. The pathological examination revealed a moderately differentiated adenocarcinoma invading from the muscularis propriae to the submucosal layer in the suture line. There was no invasion of tumor cells to either the small intestine or the surgical margins, to which direct invasion had been suspected based on the preoperative CT (Figure 4).

**Figure 5:**
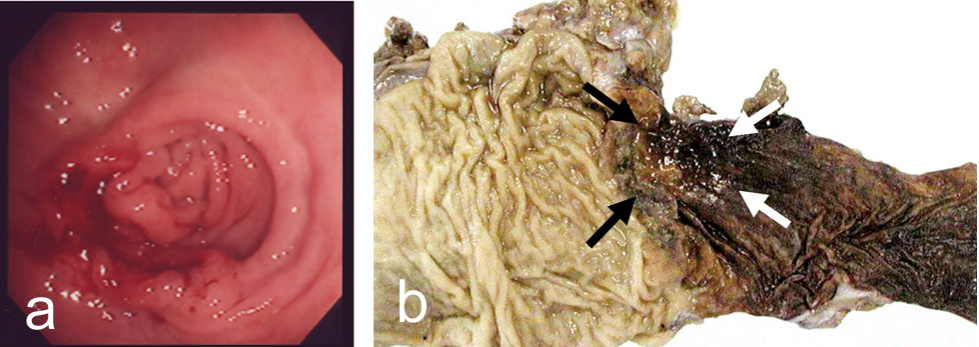
**The third anastomotic recurrence**. Colonoscopy showed a lesion with ulceration at the suture line (a). The 2.0 × 4.0 cm lesion was located in the suture line (arrow). The pathological examination revealed a moderately differentiated adenocarcinoma invading the subserosa, and there was no invasion of tumor cells to any surgical margin.

The patient noticed bleeding per rectum during defecation in July 2002, seven months after the prior operation. Romanoscope and colonoscopy showed a tumor at the suture line, and the pathological examination of the biopsy confirmed a diagnosis of adenocarcinoma. Although the serum CEA level (5.1 ng/ml) was within normal limits, the CA19-9 level was 50.4 U/ml this time. There was no metastasis in either the abdominal cavity or the distant organs by preoperative CT and US preoperatively. We performed abdominoperineal resection with lateral lymphadenectomy for the third anastomotic recurrence on September 2002. The lesion measured 2.0 by 4.0 cm, and was located in the suture line. The pathological examination revealed a moderately differentiated adenocarcinoma invading the subserosa, and there was no invasion of tumor cells to any surgical margin. Metastases of tumor cells were not identified histologically in any resected lymph nodes (Figure 5). It has now been seven years and nine months since the first operation, and there has been no further recurrence.

## Discussion

Anastomotic recurrence occurring repeatedly after curative surgery for colorectal cancer is rare. We were unable to find another report like this case, in which anastomotic recurrence had occurred repeatedly after curative surgery. Anastomotic recurrence after curative surgery of colorectal cancer may be due to several causes: implantation of free intraluminal cancer cells on a raw surface or the suture materials; instability of the mucosa at the site of an anastomosis; or positive distal margin of resection [4,5]. It is difficult to identify the cause of anastomotic recurrence exactly because of the difficulty of early diagnosis. Several researchers have reported tumor cell implantation as the mechanism of anastomotic recurrence [6-8]. In this case tumor cells were not identified histologically at the surgical margins in each of the resected specimens. Also, there was no evidence that the residual tumor cells around the tumor had invaded directly to the anastomotic site intra-operatively. Therefore, implantation of exfoliated malignant cells is suggested as a possible mechanism of anastomotic recurrence in this case. Many researchers have demonstrated that irrigation of the lumen with 5% povidone-iodine is useful to prevent anastomotic recurrence [7-10]. However, we were unable to prevent anastomotic recurrence despite irrigation with 5% povidone-iodine before anastomosis [11]. It is difficult to detect an early anastomotic recurrence. Although analysis of local recurrence in the Stockholm Rectal Cancer Trial revealed that pain was reported as the most dominant symptom, it was not always an early sign of anastomotic recurrence [12]. Although the serum CEA levels considered to be a good predictor for recurrent disease, Moertel *et al*., reported that CEA testing was most sensitive for hepatic or retroperitoneal metastasis and relatively insensitive for local, pulmonary, or peritoneal involvement [13]. In this case, CEA monitoring was not useful for early detection of anastomotic recurrence. Whichmann *et al*., also reported that CEA monitoring in which the primary tumor produced no elevation in the serum CEA level, as in this case, was not useful for the early detection of recurrent disease [14]. In this case, colonoscopy was more useful for detecting the patients with occult anastomotic recurrence compared to CEA monitoring. It is now seven years and nine months since the first operation, and there has been no further recurrence. The prognosis of this patient is good, even though anastomotic recurrence occurred repeatedly.

## Conclusion

We experienced a rare case of anastomotic recurrence that occurred three times after curative surgery for sigmoid colon cancer, and which we could not prevent by irrigation with 5% povidone-iodine. The prognosis for anastomotic recurrence is good even though anastomotic recurrence occurred three times in this case. Follow-up colonoscopy was helpful for the diagnosis of anastomotic recurrence compared with CEA monitoring.

## Competing interests

The author(s) declare that they have no competing interests.

## Authors' contributions

KF: Primary author. Carried out all the surgical procedures.

JK: A contributing author. Carried out the surgical procedures with KF.

NS, HS and KS: Literature review and report preparation.

TT: Consultant surgeon. Approved the manuscript for publication.

All authors read and approved the revised version of this manuscript for publication.
